# Cucurbitacin B and SCH772984 exhibit synergistic anti-pancreatic cancer activities by suppressing EGFR, PI3K/Akt/mTOR, STAT3 and ERK signaling

**DOI:** 10.18632/oncotarget.21704

**Published:** 2017-10-09

**Authors:** Jingkai Zhou, Tiangang Zhao, Linfeng Ma, Min Liang, Ying-Jie Guo, Li-Mei Zhao

**Affiliations:** ^1^ Department of Pharmacy, Shengjing Hospital of China Medical University, Shenyang, China; ^2^ School of Life Sciences, Jilin University, Changchun, China

**Keywords:** cucurbitacin B, SCH772984, pancreatic cancer, EGFR, ERK

## Abstract

Cucurbitacin B (CuB) is a natural tetracyclic triterpene product and displays antitumor activity across a wide array of cancers. In this study, we explored the anti-pancreatic cancer activity of CuB alone and in combination with SCH772984, an ERK inhibitor, *in vitro* and *in vivo*. CuB inhibited proliferation of pancreatic cancer cells by arresting them in the G2/M cell cycle phase. This was associated with inhibition of EGFR expression and activity and downstream signaling, including PI3K/Akt/mTOR and STAT3. Interestingly, ERK activity was markedly enhanced by activating AMPK signaling after 12 h of CuB treatment. SCH772984 potentiates the cytotoxic effect of CuB on pancreatic cancer cells through complementary inhibition of EGFR, PI3K/Akt/mTOR, STAT3 and ERK signaling, followed by an increase in the pro-apoptotic protein Bim and a decrease in the anti-apoptotic proteins Mcl-1, Bcl-2, Bcl-xl and survivin. Furthermore, combined therapy with CuB and SCH772984 resulted in highly significant growth inhibition of pancreatic cancer xenografts. These results may provide a basis for further development of combining CuB and ERK inhibitors to treat pancreatic cancer.

## INTRODUCTION

Pancreatic cancer is the fourth most common cause of cancer deaths in Western societies, and fewer than 5% of newly diagnosed patients survive more than 5 years [1]. The low survival rate is primarily due to the insensitivity of pancreatic cancer to most chemotherapy and radiotherapy treatments [1, 2]. Therefore, there is an urgent need to develop new agents or combination therapeutic strategies to treat this deadly disease.

Epidermal growth factor receptor (EGFR) plays an important role in pancreatic cancer progression [3]. EGFR is a member of the erb-B receptor tyrosine kinase (TK) family. The activated receptor can induce several downstream signaling pathways, including Ras/Raf/mitogen-activated extracellular signal-regulated kinase/extracellular signal-regulated kinase (Ras/Raf/MEK/ERK), phosphatidylinositol 3-kinase/viral Akt homologue/mammalian target of rapamycin (PI3K/Akt/mTOR), and signal transducer and activator of transcription 3 (STAT3). These pathways effectively promote cell proliferation, invasion and metastasis by modulating expression of both pro- and anti-apoptotic genes, such as the Bcl-2 family of proteins or survivin [4, 5]. EGFR overexpression has been observed in 30%–89% of pancreatic cancers [6, 7] and is associated with advanced disease, poor survival and metastasis [8]. Thus, EGFR has been considered a potential target for treating pancreatic cancer. However, due to a high rate of acquired or inherent resistance, EGFR inhibitors are insufficient in effectively treating human pancreatic cancer [9]. The most well defined mechanisms of resistance are involved in EGFR-independent constitutive activation of downstream effectors, such as mitogen-activated protein kinases (MAPK) [10], PI3K [11] and STAT3 [11], or complex crosstalk among EGFR downstream signaling pathways [12, 13]. Preclinical studies evaluating combination therapy with EGFR and RAS pathway inhibitors in pancreatic cancer have shown promising results [14–16]. Therefore, blocking EGFR and its downstream signaling targets might be a rational strategy for pancreatic cancer therapy.

Cucurbitacins are natural tetracyclic triterpene compounds derived from plants in the Cucurbitaceae family and have a wide spectrum of pharmacological activities, such as anticancer, anti-inflammatory and hepatoprotective effects [17]. Cucurbitacin B is one of the most abundant forms of cucurbitacins and has received increasing attention in recent years for its cytotoxicity across a wide array of cancers, including pancreatic cancer [18–20]. STAT3 is the primary therapeutic target of CuB in pancreatic cancer. CuB induces pancreatic cancer cell apoptosis through inhibition of STAT3 signaling [21–26]. Interestingly, accumulated data have demonstrated that CuB can selectively inhibit other signaling pathways dependent on cancer cell contexts. For example, CuB suppressed the expression and activity of HER2 as well as EGFR in HER2-overexpressed breast cancer cells [27]. Silva et al. demonstrated that a CuB derivate had strong cytotoxicity in EGFR-overexpressed non-small-cell lung cancer by interfering with EGFR activation and its downstream effectors, Akt, ERK and STAT3 [28]. Pancreatic cancer is an EGFR-overexpressed solid tumor. Thus, we investigated whether CuB inhibits pancreatic cancer cell growth by modulating EGFR and its downstream signaling targets and tested whether CuB in combination with downstream EGFR signaling enhances its therapeutic efficacy.

The results of the present study demonstrate that CuB treatment alone had strong growth inhibitory effects in pancreatic cancer cells through inhibition of EGFR expression level and activity. Interestingly, the PI3K/Akt/mTOR pathway and STAT3 activity were potently downregulated after 6 h of CuB treatment, whereas ERK activity was markedly upregulated after 12 h of CuB treatment by enhancing AMPK activity. Because ERK plays crucial roles in tumorigenesis, cell proliferation and inhibition of apoptosis [29], we infer that reactivation of ERK may be a potent mechanism for resistance to CuB efficacy and hypothesize that direct inhibition of ERK activation may overcome this limitation. SCH772984, a selective inhibitor of ERK1/2 that has been shown to act as type I and type II kinase inhibitors and inhibit tumor proliferation in mouse pancreatic cancer and breast cancer xenograft models [30, 31], was selected to evaluate its effect on CuB-induced cytotoxicity. Synergistic antitumor interactions between CuB and SCH772984 were observed *in vitro* and *in vivo*.

## RESULTS

### CuB inhibits proliferation of pancreatic cancer cells

**Figure 1:**
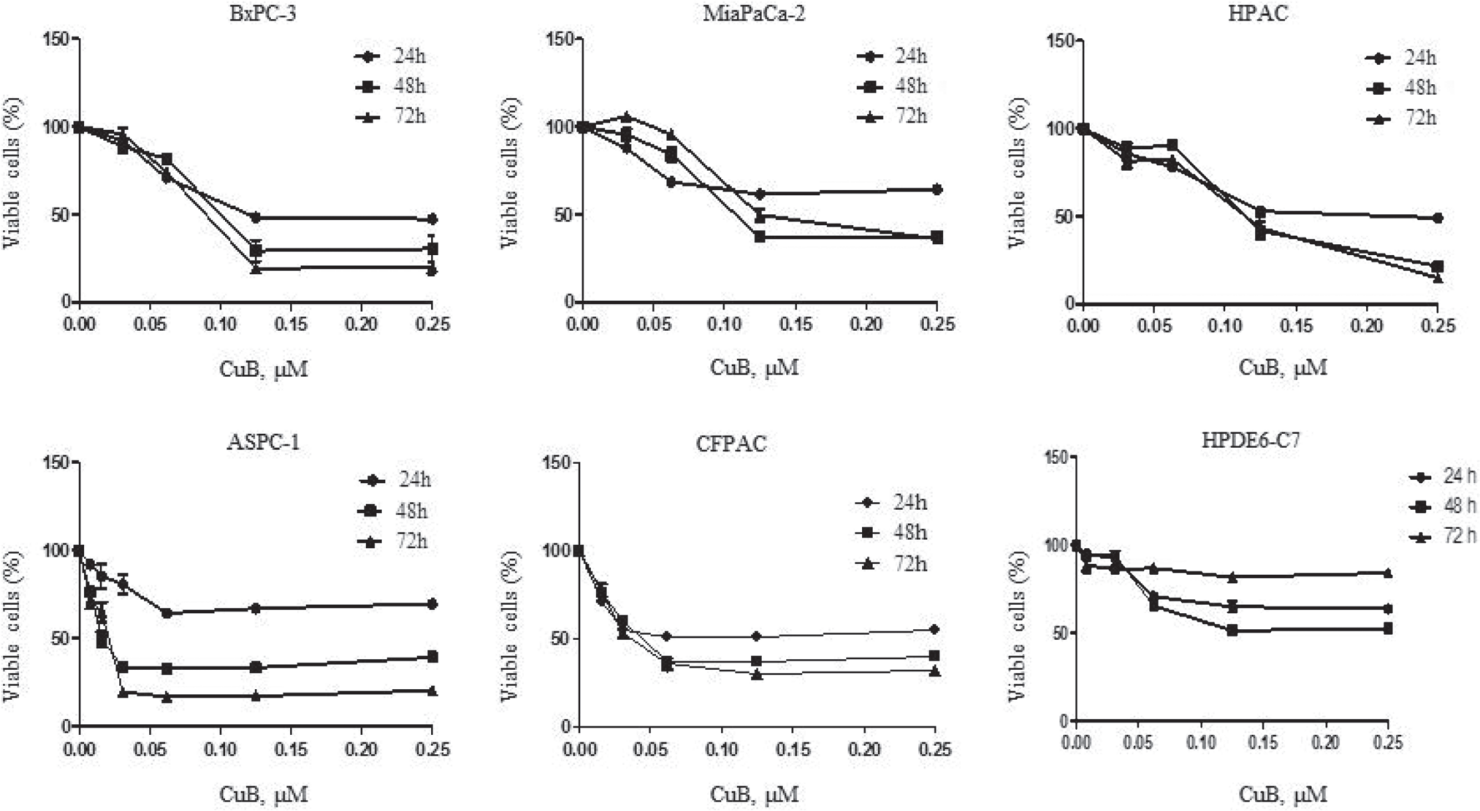
CuB inhibits proliferation of pancreatic cancer cells BxPC-3, MiaPaCa-2, HPAC, ASPC-1, CFPAC and HPDE6-C7 cells were cultured with CuB for 24, 48, and 72 hours. Viable cells were determined with the MTT assay. Data are presented as the mean ± standard error from at least 3 independent experiments.

Considering pancreatic cancer cell heterogeneity, we tested the ability of CuB to inhibit proliferation in 5 pancreatic cancer cell lines (BxPC-3, MiaPaCa-2, HPAC, CFPAC and ASPC-1) via the MTT assay. A normal pancreatic ductal epithelial cell line (HPDE6-C7) was used as a control. Because CuB is dissolved in less than 1%. DMSO in cell culture medium, we first observed the effect of DMSO on cell proliferation. MTT analysis revealed that different concentrations of DMSO ranging from 1/300,000 to 1/1,000 did not significantly inhibit proliferation in all cell lines evaluated (Supplementary Figure 1). Interestingly, CuB potently inhibited proliferation in all pancreatic cancer cell lines in dose- and time-dependent manners (Figure 1). IC_50_ values varied, ranging from 0.017 μM (AsPC-1) to 0.278 μM (MiaPaCa-2) after 72 h of treatment. CuB did not have a significant effect on normal pancreatic ductal epithelial cell proliferation after 72 h of treatment (Figure 1). This indicates that CuB has stronger growth inhibitory effects on pancreatic cancer cells compared to normal pancreatic ductal epithelial cells after 72 h of treatment.

### CuB leads to cell cycle arrest and cell death

**Figure 2:**
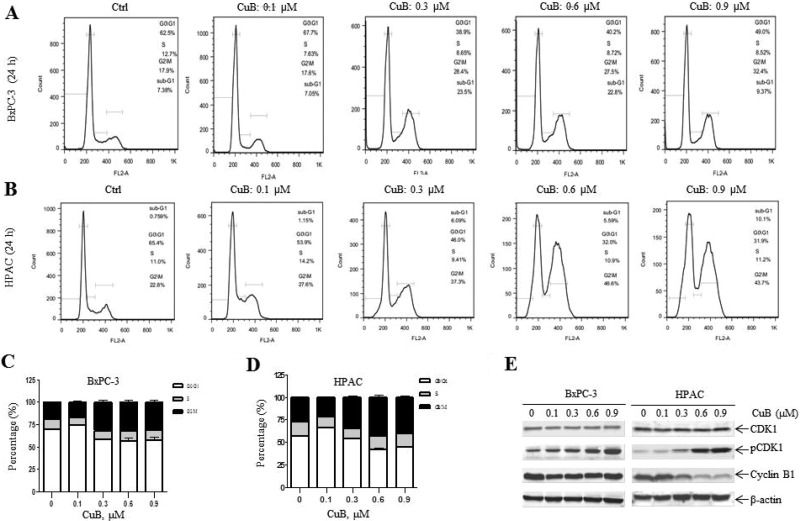

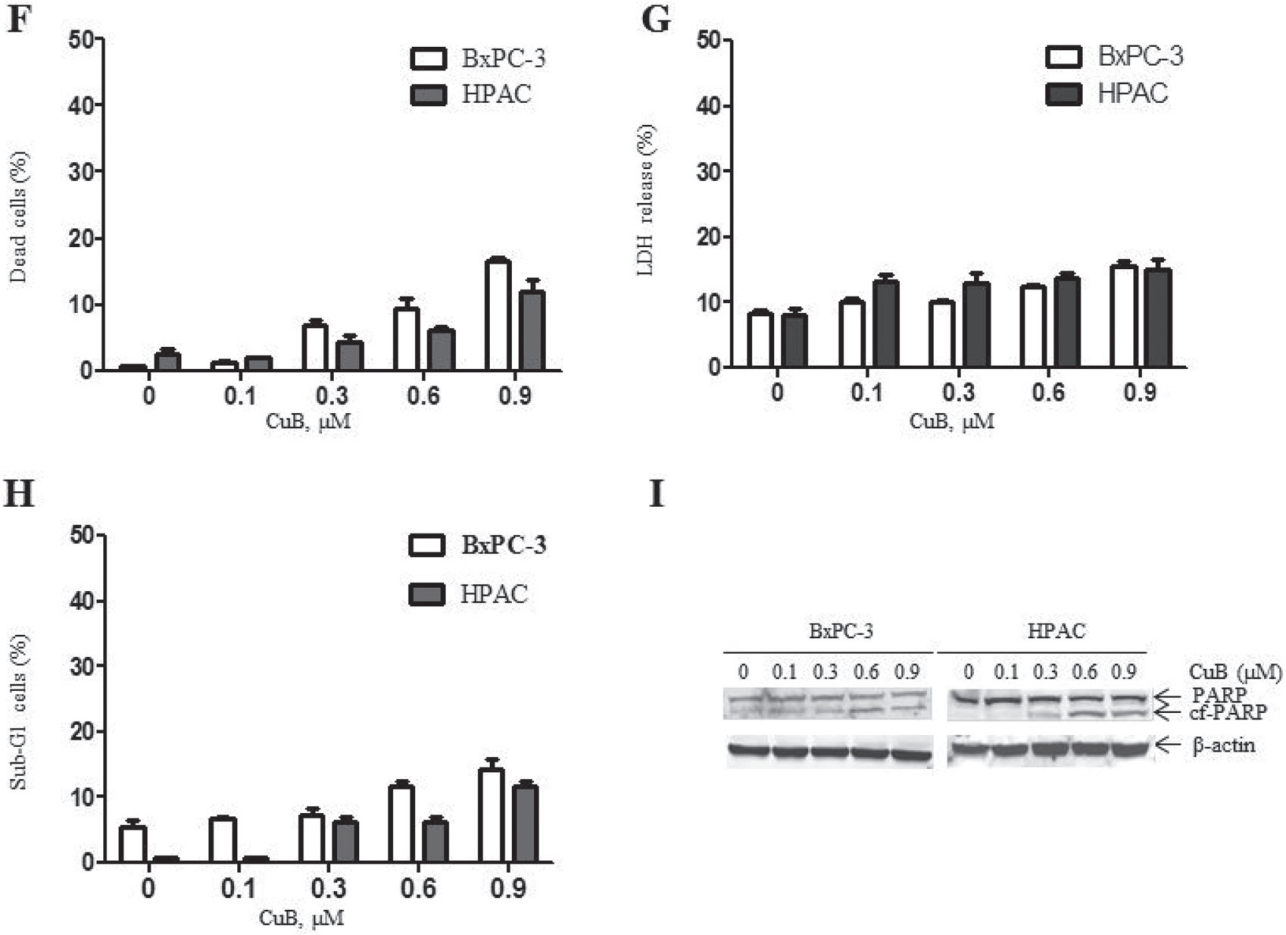
CuB leads to cell cycle arrest and cell death (**A** and **B**) BxPC-3 and HPAC cells were treated with vehicle control or varying concentrations of CuB for 24 h, fixed with 80% ice-cold ethanol, and stained with PI for cell cycle analysis. Representative histograms are shown. (**C** and **D**) Histograms represent the relative distribution of non-apoptotic cells between the G0/G1, S, and G2/M phases. (**E**) Whole cell lysates were analyzed by Western blotting and probed with anti-CDK1, -pCDK1, -Cyclin B1, or -β-actin antibodies. (**F**) BxPC-3 and HPAC cells were treated with vehicle control or different CuB concentrations for 24 h, then stained with trypan blue. Histograms represent the percentage of dead cells. (**G**) BxPC-3 and HPAC cells were treated with vehicle control or different CuB concentrations for 24 h. Culture medium was collected and LDH release was assessed. Histograms represent the percentage of LDH release. (**H**) Sub-G1 data are presented as the mean of triplicate data ± SEM from one representative experiment. (**I**) BxPC-3 and HPAC cells were treated with vehicle control or different CuB concentrations for 24 h. Whole cell lysates were analyzed by Western blotting and probed with anti-PARP or -β-actin antibodies. Experiments were performed at least 3 independent times, and representative Western blots are shown.

To explore the growth inhibitory mechansims of CuB, we treated 2 pancreatic cancer cell lines that have different Ras and p53 phenotypes [BxPC-3 (Ras wild-type, p53 mutation) and HPAC (Ras mutation, p53 wild-type)] with varying concentrations of CuB for 24 h. Cell cycle distribution was observed by PI staining followed by flow cytometry. CuB induced cell accumulation in the G2/M phase in a dose-dependent manner, accompanied by a decrease in the G0/G1 phase fraction (Figure 2A–2D). We also observed expression of G2/M phase-related proteins by Western blotting analysis. CuB treatment resulted in an increase in Tyr15 phosphorylated CDK1 in BxPC-3 and HPAC cells (Figure 2E). Cyclin B1 protein levels remained unchanged in BxPC-3 cells, but were decreased in HPAC cells (Figure 2E). This indicates that CuB inhibits pancreatic cancer cell growth by arresting cells in the G2/M cell cycle phase.

Another mechanism of cell growth inhibition is to cause cell death. Trypan blue exclusion analysis showed that less than 15% of cells were observed to be dead at the highest concentration of 0.9 µM in both cell types (Figure 2F), indicating that CuB alone has a limited ability to cause cell death, which was further demonstrated by LDH release assay (Figure 2G). A low number of cells with DNA fragments (Sub-G1) was also observed after CuB 24 h treatment (Figure 2H), accompanied by an increase in cleaved PARP (Figure 2I).

### CuB suppresses EGFR and downstream PI3K/Akt/mTOR and STAT3 signaling

**Figure 3:**
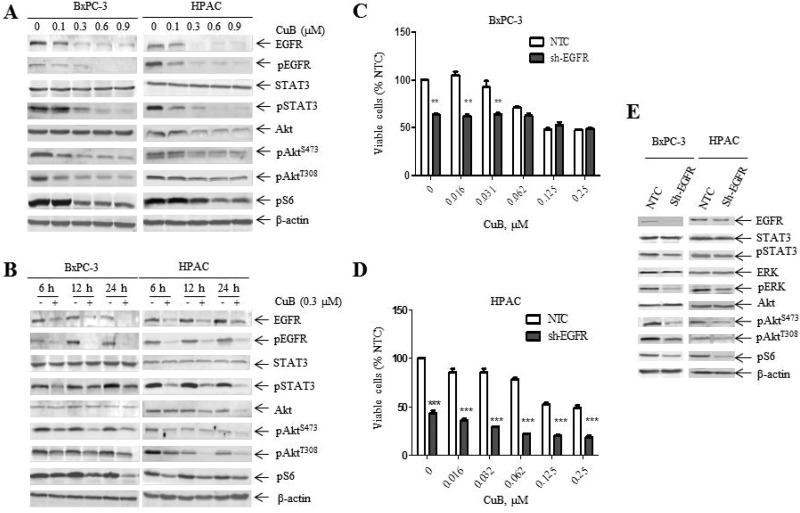
CuB suppresses EGFR levels and downstream PI3K/Akt/mTOR and STAT3 signaling (**A**) BxPC-3 and HPAC cells were treated with vehicle control or CuB for 24 h. Whole cell lysates were analyzed by Western blotting and probed with the indicated antibodies. (**B**) BxPC-3 and HPAC cells were treated with 0.3 µM CuB for up to 24 h. Cells were harvested and lysed. Protein extracts were analyzed by Western blotting and probed with the indicated antibodies. (**C** and **D**) BxPC-3 and HPAC cells were infected with EGFR (EGFR-shRNA) or non-target control shRNA lentivirus (NTC-shRNA). EGFR-shRNA or NTC-shRNA groups were cultured with CuB for 24 h. Cell viability was determined daily using the MTT assay. Data are presented as the mean ± standard error from at least 3 independent experiments. Statistical significance was calculated using the pair-wise 2-sample *t*-test. ^***^indicates *p* < 0.001, ^**^ *p* < 0.01. (**E**) NTC- and EGFR-shRNA whole cell lysates were analyzed by Western blotting and probed with the indicated antibodies. Experiments were performed at least 3 independent times, and representative Western blots are shown.

We examined the association between the growth inhibitory effect of CuB and EGFR signaling in BxPC-3 and HPAC cells. CuB effectively inhibited EGFR and pEGFR levels in both cell types in a dose-dependent manner (Figure 3A). CuB also reduced the levels of the EGFR downstream effectors pAkt (T308), pAkt (S473), pS6 and pSTAT3 without altering total protein levels of Akt and STAT3 in BxPC-3 cells (Figure 3A). Similar results were also observed in HPAC cells, except for decreased Akt levels (Figure 3A). We then used 0.3 μM CuB to determine the effects over time. In both cell lines, decreased EGFR, pEGFR, pAkt (T308), pAkt (S473), pS6 and pSTAT3 levels were detected as early as 6 h and remained low levels throughout the 24 h time period (Figure 3B). This indicates that EGFR, PI3K/Akt/mTOR and STAT3 signaling are simultaneously inhibited after 6 h of CuB treatment.

To provide further evidence that decreased EGFR levels are required for CuB-induced growth inhibition in pancreatic cancer cells, we performed lentiviral shRNA knockdown of EGFR in BxPC-3 and HPAC cells (EGFR-shRNA), which was compared to non-target control cells (NTC-shRNA) by Western blotting analysis (Figure 3E). Cell proliferation in the absence or presence of CuB was then determined by the MTT assay. EGFR-shRNA cells had a slower growth rate than NTC-shRNA cells in the absence of CuB (Figure 3C and 3D). CuB significantly inhibited the growth of EGFR-shRNA HPAC cells at all concentrations evaluated compared to NTC-shRNA HPAC cells, whereas the growth of EGFR-shRNA BxPC-3 cells was significantly inhibited compared to NTC-shRNA BxPC-3 cells at low CuB concentrations (Figure 3C and 3D). We also observed decreased protein levels of the EGFR downstream effectors pAkt (T308), pAkt (S473), pS6, pSTAT3 and pERK in EGFR-shRNA cells compared to NTC-shRNA cells without changes in total protein levels (Figure 3E). This suggests that downregulation of EGFR protein levels is responsible for the growth inhibitory effect of CuB in pancreatic cancer cells.

### CuB enhances ERK activity by activating AMPK signaling

**Figure 4:**
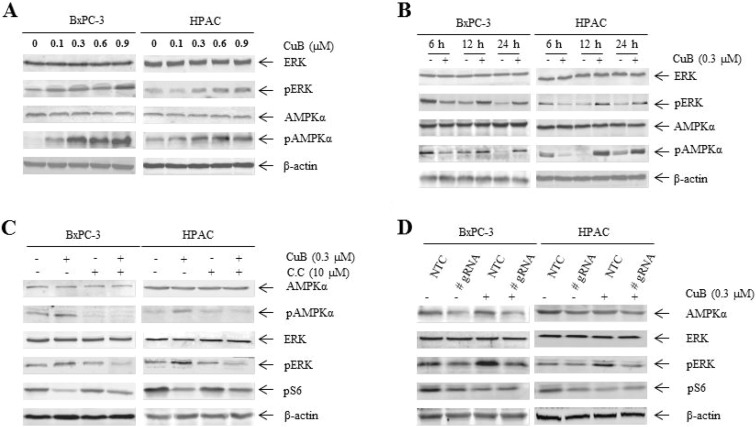
CuB enhances ERK activity via AMPK activation (**A**) BxPC-3 and HPAC cells were treated with vehicle control or CuB for 24 h. Whole cell lysates were analyzed by Western blotting and probed with anti-AMPKα, -pAMPKα, -ERK, -pERK or -β-actin antibodies. (**B**) BxPC-3 and HPAC cells were treated with 0.3 µM CuB for up to 24 h. Cells were harvested and lysed. Protein extracts were analyzed by Western blotting and probed with the indicated antibodies. (**C**) BxPC-3 and HPAC cells were treated with CuB and compound C (C.C) alone or in combination for 24 h. Whole cell lysates were analyzed by Western blotting and probed with anti-AMPKα, -pAMPKα, -ERK, -pERK, -pS6 or -β-actin antibodies. (**D**) BxPC-3 and HPAC cells were infected with non-template control (NTC) or AMPKα CRISPR lentivirus (#gRNA). Cells were then treated with or without CuB for 24 h. Whole cell lysates were analyzed by Western blotting and probed with the indicated antibodies. Experiments were performed at least 3 independent times, and representative Western blots are shown.

Since the MEK/ERK pathway is also an important downstream component of EGFR signaling, we next observed the effect of CuB on ERK activity. There was a dose-dependent stimulatory effect on ERK activity after 24 h of CuB treatment in BxPC-3 and HPAC cells, as monitored by ERK phosphorylation (Figure 4A). Time course experiments revealed a dynamic change in pERK levels during 24 h of CuB treatment in both cell lines. Upon CuB treatment, pERK levels were decreased at 6 h, then increased after 12 h relative to the corresponding vehicle control treatment (Figure 4B).

We observed decreased pERK levels in EGFR-shRNA cells relative to NTC-shRNA control cells (Figure 3G), suggesting that enhanced pERK levels may be independent of EGFR downregulation by CuB. Given that potential cross-talk between AMPK and ERK signaling has been reported [32–34], we examined phosphorylated and total protein levels of AMPKα after 24 h of CuB treatment. CuB effectively enhanced pAMPKα levels in both cell lines in a dose-dependent manner without altering total AMPKα protein levels (Figure 4A). Importantly, time course experiments also showed decreased pAMPKα levels at 6 h and increased pAMPKα levels after 12 h relative to the corresponding vehicle control treatment (Figure 4B). Phosphorylated levels of AMPKα and ERK showed corresponding changes over time in both cell lines. We observed the effects of compound C, a selective AMPK inhibitor, on pERK levels using Western blotting analysis. Compound C significantly inhibited an increase in pERK levels by CuB in both cell lines (Figure 4C), suggesting that CuB may increase pERK protein levels by activating AMPK. Because AMPK negatively regulates the mTOR signaling pathway, we also observed pS6 levels in the presence of CuB and compound C. Decreased pS6 levels due to CuB were restored to some extent after the combined treatment (Figure 4C). Consistently, AMPKα CRISPR knockdown reversed the increase in pERK and decrease in pS6 levels due to CuB in BxPC-3 and HPAC cells (Figure 4D). These results demonstrate that AMPK activation plays an important role in CuB-induced ERK phosphorylation and pS6 downregulation.

### SCH772984 synergizes with CuB to induce growth inhibition and apoptosis of pancreatic cancer cells

**Figure 5:**
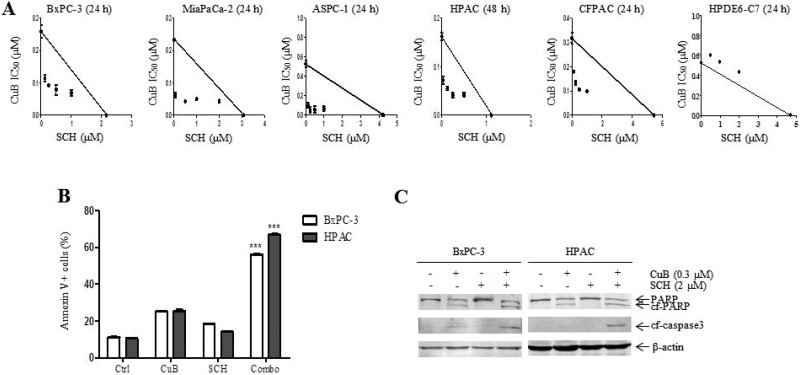
SCH772984 synergizes with CuB to induce growth inhibition and apoptosis of pancreatic cancer cells (**A**) Standard isobologram analyses of antitumor interactions between SCH772984 and CuB in pancreatic cancer cells were performed. (**B**) BxPC-3 and HPAC cells were treated with vehicle control, CuB (0.3 µM), SCH772984 (2 µM) or CuB plus SCH772984 for 24 h. Cell death was determined by annexin V/PI staining and flow cytometry analyses. Dead cells are expressed as the percentage of annexin V+ cells. Data are presented as the mean of triplicate experiments ± standard error from 1 representative experiment. Statistical significance was calculated using the pair-wise 2-sample *t*-test. ^***^indicates *p* < 0.001. (**C**) Protein extracts were analyzed by Western blotting and probed with anti-PARP, -cf-caspase3 or -β-actin antibodies.

To explore the effect of ERK over-activation on CuB-induced cytotoxicity, cell proliferation was measured after 24 h or 48 h of treatment with CuB and SCH772984 alone or in combination using the MTT assay. When administered simultaneously, SCH772984 significantly enhanced sensitivity to CuB, which is reflected by decreased IC_50_ values in 5 pancreatic cancer cell lines (Figure 5A and Table 1). Combined effects of CuB with SCH772984 on growth of the 5 pancreatic cancer cells were clearly synergistic, as shown by all points below the line using standard isobologram analysis and all CIs (combination indices) < 1.00 (Figure 5A and Table 1). In contrast, combined effects of CuB with SCH772984 on normal pancreatic ductal epithelial cell proliferation were antagonistic, shown as all points above the line using standard isobologram analysis and all CIs > 1.00. These data indicate that SCH772984 significantly enhanced CuB sensitivity in pancreatic cancer cells, but not in normal pancreatic ductal epithelial cells.

**Table 1:** Effects of SCH772984 on CuB sensitivities in pancreatic cell lines

Cell lines	IC50 of SCH772984 (μM)	IC50 of CuB (μM) in the absence or presence of SCH772984 (μM)				*P* Value
		0	0.5	1	2	
BxPC-3 ASPC-1 MiaPaCa-2 HPAC CFPAC HPDE6-C7	2.41 ± 0.78 4.23 ± 0.66 3.07 ± 0.34 1.12 ± 0.16 5.42 ± 0.77 4.72 ± 0.86	0.20 ± 0.01 0.14 ± 0.06 0.25 ± 0.02 0.18 ± 0.05 0.32 ± 0.01 0.51 ± 0.04	0.11 ± 0.04 (0.66) 0.11 ± 0.09 (0.08) 0.16 ± 0.02 (0.11) 0.06 ± 0.01 (0.26) 0.25 ± 0.12 (0.32) 0.50 ± 0.26 (> 1)	0.09 ± 0.03 (0.71) 0.12 ± 0.09 (0.13) 0.17 ± 0.02 (0.16) 0.04 ± 0.01 (0.48) 0.28 ± 0.13 (0.20) 0.51 ± 0.13 (> 1)	0.07 ± 0.02 (0.67) 0.12 ± 0.08 (0.13) 0.18 ± 0.03 (0.16) 0.04 ± 0.01 (0.21) 0.23 ± 0.14 (0.58) 0.48 ± 0.14 (> 1)	< 0.05 < 0.05 < 0.05 < 0.05 < 0.05 > 0.05

Note: CuB and SCH772984 IC_50_s are presented as the mean ± standard error from at least three independent experiments. Numbers in parentheses represent the combination index values. *P* values for each pair were determined using GraphPad Prism 5.0.

We next investigated whether CuB and SCH772984 could cooperate to induce cell death. Annexin V/PI double staining and flow cytometry analyses showed that combination treatment significantly increased cell apoptosis relative to individual treatment in BxPC-3 and HPAC cells (Figure 5B), which was accompanied by increased PARP and caspase-3 cleavage (Figure 5C). These results demonstrate that CuB and SCH772984 synergistically promote pancreatic cancer cell apoptosis.

### CuB in combination with SCH772984 inhibits EGFR and downstream signaling

**Figure 6:**
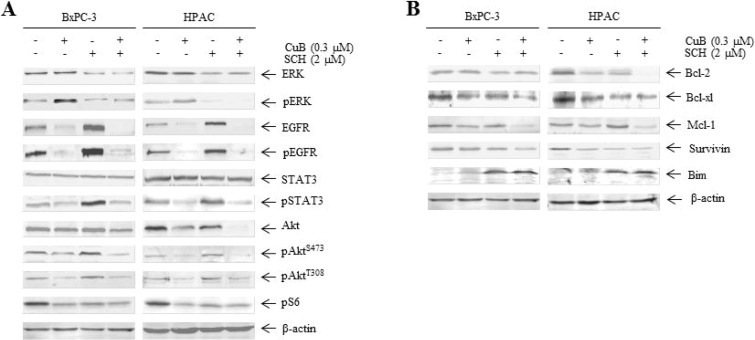
CuB in combination with SCH772984 inhibits EGFR and downstream signaling (**A** and **B**) BxPC-3 and HPAC cells were treated with CuB and SCH772984 alone or in combination for 24 h. Whole cell lysates were analyzed by Western blotting and probed with the indicated antibodies.

We observed the effects of CuB in combination with SCH772984 on EGFR and downstream signaling. SCH772984 at 2 μM abrogated both ERK protein levels and ERK phosphorylation, which is in accordance with its effect as an ERK inhibitor. As expected, CuB-activated ERK phosphorylation was markedly suppressed by combination treatment with SCH772984 and CuB (Figure 6A). In addition, EGFR, pEGFR, pAkt (T308), pAkt (S473), pS6 and pSTAT3 levels were lower after the combined treatment than after vehicle control treatment in BxPC-3 and HPAC cells (Figure 6A). We also investigated the effects of SCH772984 and CuB, either alone or in combination, on Bcl-2 family proteins and survivin in both cell types. Mcl-1 protein levels were much lower after the combined treatment than after CuB or SCH772984 treatment alone. The combined treatment also resulted in an increase in Bim levels and a decrease in Bcl-2, Bcl-xl and survivin compared to vehicle control (Figure 6B).

### Antitumor efficacy of CuB and SCH772984 *in vivo*

**Figure 7:**
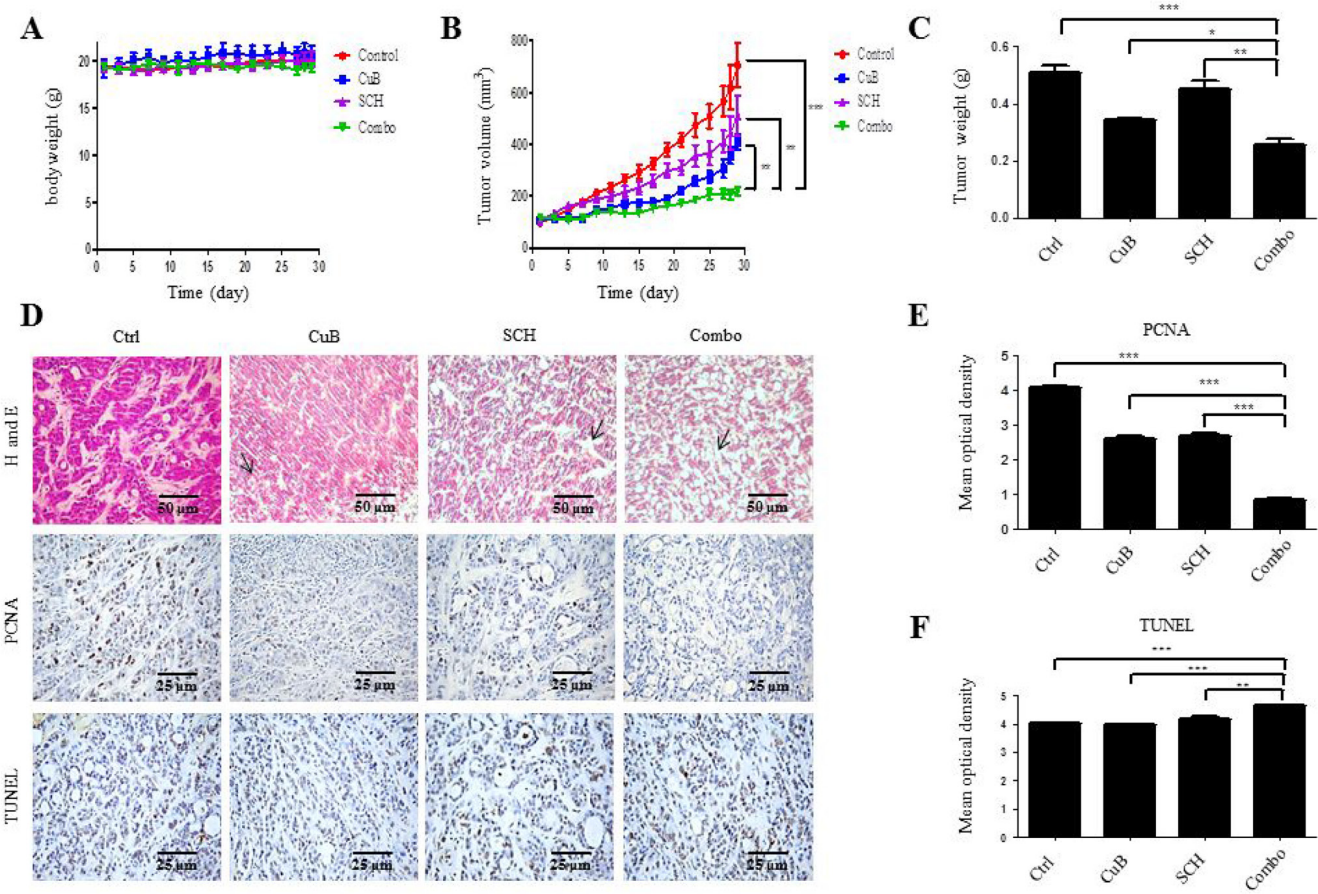
Antitumor efficacy of CuB and SCH772984 *in vivo* (**A**) Body weights were measured daily. (**B**) Tumor volumes were calculated according to the following formula: m_1_^2^ × m_2_× 0.5236 (m_1_: short diameter; m_2_: long diameter). (**C**) Overall weight of the dissected tumors. Mean ± SD of overall tumor weight was measured at autopsy. (**D**) HE and immunohistochemical staining were evaluated by microscopy at ×200 magnification, scale bar = 50 μm. (**E**) Immunohistochemical staining and (**F**) TUNEL were analyzed with Image Motic Images Advanced 3.2, scale bar = 25 μm. Graphed as the mean ± standard error. ^***^p < 0.001, ^**^ *p* < 0.01, ^*^ *p* < 0.05.

We used a mouse HPAC xenograft model to evaluate the effects of CuB and SCH772984 on pancreatic tumor growth. Individual and combined drug treatments were well tolerated, as indicated by the lack of a significant loss of body weight (Figure 7A). Compared to vehicle control treatment, successive 4-week treatment with CuB or SCH772984 alone significantly reduced tumor growth, resulting in lower mean tumor volumes (63.8% and 54.7% on day 28, respectively, Figure 7B). In particular, the combined drug treatment resulted in significant delay of tumor growth during the treatment period compared to single drug treatment, with 85.0% tumor growth inhibition on day 28 (Figure 7B). Decrease in tumor volume was also verified by a decrease in tumor weight (Figure 7C). CuB in combination with SCH772984 significantly decreased the average tumor weight relative to CuB or SCH772984 alone on day 28.

To further investigate the *in vivo* effects of CuB and SCH772984 treatment, tumors were analyzed by HE, immunohistochemical and TUNEL staining. Individual drug treatment resulted in increased tumor necrosis, which was further increased following combination treatment, as indicated in arrows in HE staining (Figure 7D). Proliferation was substantially lower in the combination group compared to the single groups, as indicated by lower PCNA staining (Figure 7E). The combined drug treatment resulted in increased cell apoptosis, as measured by the TUNEL assay (Figure 7F). These data emphasize the potential for using combined CuB and SCH772984 to treat pancreatic cancer.

## DISCUSSION

**Figure 8:**
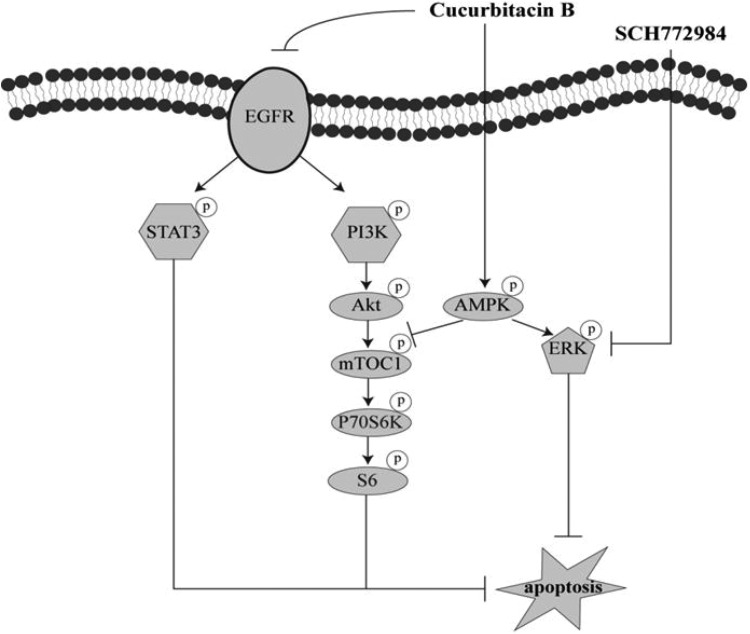
Proposed model of synergistic anti-pancreatic cancer activities of CuB and SCH772984 CuB suppresses EGFR levels, activity and downstream PI3K/Akt/mTOR and STAT3 signaling, but enhances ERK activity via AMPK activation, which inactivates the canonical apoptosis pathway. Addition of SCH772984 reverses ERK phosphorylation induced by CuB, thereby resulting in apoptosis.

Pancreatic cancer is an oncogene-driven tumor with multiple genetic and epigenetic alterations. These genetic alterations may contribute to its aggressive nature and confer resistance to conventional and targeted agents [35, 36]. EGFR overexpression has been observed in most pancreatic cancer patients [6, 8] and plays prominent roles in malignant transformation, prevention of apoptosis, and drug resistance [3]. Due to a high rate of acquired or inherent resistance, targeting EGFR has proven to be insufficient in effectively treating human pancreatic cancer [9]. In addition, targeting one downstream pathway usually leads to compensatory activation of interconnected survival pathways. In this study, we demonstrate for the first time that CuB inhibits pancreatic cancer cell proliferation by interfering with EGFR levels and downstream signaling of PI3K/Akt/mTOR and STAT3. CuB enhances ERK activity via activating AMPK signaling (Figure 8). The ERK-selective inhibitor SCH772984 synergistically enhanced CuB-induced cell death in 5 pancreatic cancer cell lines (Figure 8). Combined treatment resulted in 85% tumor growth inhibition on day 28 in a HPAC xenograft mouse model.

In this study, we selected 5 pancreatic cancer cell lines with different genetic backgrounds to observe the effects of CuB on cell growth. CuB inhibited pancreatic cancer cell growth with IC_50_ values between 0.017 μM (ASPC-1) and 0.278 μM (MiaPaCa-2) after 72 h treatment. This indicates that CuB has strong growth inhibitory effects in pancreatic cancer cells, with all IC_50_ values below 1 μM. Both MiaPaCa-2 (insensitive to CuB) and ASPC-1 (sensitive to CuB) have Ras and p53 mutations, which occur in 90% and 50–75% of pancreatic cancer patients, respectively, and can activate multiple signaling pathways related to cell proliferation and anti-apoptosis [37, 38]. This suggests that CuB-induced growth inhibitory effects are not associated with Ras and p53 mutations in pancreatic cancer cells. It has been reported that CuB can inhibit pancreatic cancer cell growth by arresting cells in the G2/M cell cycle phase [21], which is consistent with our results. Another mechanism of cell growth inhibition is to cause cell death. Interestingly, CuB at the highest concentration of 0.9 µM only led to about 15% cell death after 24 h of treatment in BxPC-3 and HPAC cells. These findings suggest that growth inhibition mainly contributes to the cytotoxicity of CuB in pancreatic cancer cells. Interestingly, 72 h of CuB treatment had fewer growth inhibitory effects on the 5 pancreatic cancer cell lines evaluated than on HPDE6-C7 cells, indicating that CuB has low toxicity in normal pancreatic ductal epithelial cells.

Inhibition of the JAK/STAT3 pathway has been classically known as a mechanism of pancreatic cancer cytotoxicity caused by CuB [22]. Our study demonstrated that CuB not only decreased pSTAT3 protein levels, but also inhibited endogenous EGFR, pEGFR, pAkt (T308), pAkt (S473) and pS6 levels in a dose-dependent manner in pancreatic cancer cells. This suggests that STAT3 is not a unique therapeutic target in pancreatic cancer cells. In addition to cytokine receptors (e.g., JAKs), STAT3 signaling can also be activated by growth factor receptor tyrosine kinases (e.g., EGFR). To elucidate an association between decreased EGFR/pEGFR and pSTAT3, we observed protein levels over time. The results showed that both EGFR/pEGFR and pSTAT3 levels were synchronously decreased as early as 6 h after CuB treatment. Further, EGFR knockdown showed that pSTAT3 levels were lower in EGFR-shRNA pancreatic cancer cells compared to NTC-shRNA control cells without altering total protein levels, suggesting that STAT3 phosphorylation is partly dependent on EGFR protein levels. Similar results were also observed in the association between decreased EGFR/pEGFR and decreased pAkt or pS6. These results indirectly demonstrate that CuB inhibits the activities of STAT3, Akt and S6 in part by downregulating EGFR expression and activity.

Fujita et al. reported that EGFR overexpression was associated with poor prognosis and tumor aggressiveness in pancreatic cancer [39]. Consistent with these findings, we demonstrated that EGFR knockdown inhibited the growth of pancreatic cancer cells. After treatment with CuB, growth of EGFR-shRNA HPAC cells was further inhibited. Interestingly, CuB had no obvious dose-dependency regarding growth inhibitory effects in EGFR-shRNA BxPC-3 cells (Figure 3C). This may be attributed to more efficient EGFR knockdown in BxPC-3 cells, as shown in Figure 3E. In addition, we also showed that treatment with 0.062, 0.125 and 0.250 μM of CuB did not result in any differences in cell viability between EGFR-shRNA and NTC-shRNA BxPC-3 cells, which may be associated with significant downregulation of EGFR expression after treatment with a high dose of CuB in BxPC-3 cells. These results suggest that EGFR may be an important target of CuB in pancreatic cancer therapy.

ERK is another major downstream effector of EGFR. Navas et al. reported that EGFR positively regulates ERK phosphorylation in pancreatic cancer cells [11], which is consistent with our EGFR knockdown results. In contrast to decreased EGFR levels and activity, pERK levels were increased in a dose-dependent manner after CuB treatment for 24 h in BxPC-3 and HPAC cells, suggesting that enhanced pERK levels may be independent of EGFR downregulation by CuB. We also observed increased pAMPKα levels following 24 h of CuB treatment in a dose-dependent manner. In particular, ERK and AMPKα showed synchronous changes in phosphorylation levels over time. Blocking AMPK function with an AMPK inhibitor or AMPK knockdown reversed the stimulatory effect of CuB on ERK activity. These findings reveal that CuB-induced ERK phosphorylation is dependent on activation of AMPK. Several studies have explored cross-talk between AMPK and ERK signaling, but the findings have been contradictory. Consistent with our results, two AMPK activators increased ERK activity in melanoma cells by inducing degradation of dual-specific phosphatase (DUSP) 6 [33]. A recent report [32] showed that 2-deoxyglucose suppressed ERK phosphorylation by LKB1/AMPK signaling. Regulation of ERK signaling by AMPK is highly complex and additional studies are needed to explain the precise mechanisms that link the two signaling pathways.

ERK is persistently activated in pancreatic cancer and plays crucial roles in tumorigenesis, cell proliferation and inhibition of apoptosis [29, 40]. Thus, ERK inhibition by MEK/ERK inhibitors is an attractive therapeutic strategy in treating pancreatic cancer. Early trials with MEK inhibitors showed clinical activity, but their efficacy was mostly limited by toxic side effects, such as skin rash and visual disturbances [41]. EGFR or STAT3 feedback activation in response to MEK inhibitors was also shown to limit efficacy in pancreatic cancer cells [12, 42]. Combination therapy with MEK and STAT3/EGFR inhibitors has been demonstrated to exert significant anti-pancreatic cancer efficacy [12, 42]. Interestingly, our results showed a decrease in EGFR, STAT3, Akt and S6 activities and an increase in ERK activity in 2 CuB-treated pancreatic cancer cell lines. We deduce that the combination treatment with CuB and an ERK inhibitor against pancreatic cancer may be more effective than single drug treatment due to complementary effects on STAT3, ERK and EGFR activities. While combined treatment with CuB and SCH772984 showed synergistic growth inhibitory effects in 5 pancreatic cancer cells, an antagonistic effect was observed in normal pancreatic ductal epithelial cells, indicating that combined anti-pancreatic cancer therapy may be effective and safe. Moreover, combination treatment with CuB and SCH772984 significantly promoted pancreatic cancer cell apoptosis by simultaneously suppressing EGFR, STAT3, ERK, Akt and S6 activities, followed by an increase in the pro-apoptotic protein Bim and a decrease in anti-apoptotic proteins Mcl-1, Bcl-2, Bcl-xl and survivin. Importantly, synergistic antitumor reactions between CuB and SCH772984 were also observed in HPAC xenograft mice. These results are in accordance with a previous report, indicating that successful treatment of pancreatic cancer may require compound inhibition of at least 4 distinct signaling cascades, including those driven by KRAS, EGFR, PI3K and STAT3 [11].

In conclusion, CuB treatment had a strong growth inhibitory effect in pancreatic cancer cells by decreasing EGFR levels and downstream signaling of PI3K/Akt/mTOR and STAT3. However, CuB increases ERK activity by activating AMPK signaling. CuB synergizes with SCH772984 to cause growth inhibition and apoptosis of pancreatic cancer cells *in vitro* and *in vivo*. This combination treatment is associated with complementary inhibition of EGFR, STAT3, ERK and PI3K/Akt/mTOR signaling, concomitant with an increase in Bim and a decrease in Mcl-1, Bcl-2, Bcl-xl and survivin. These data suggest that the activated AMPK-ERK pathway may contribute to resistance of pancreatic cancer cells to CuB.

## MATERIALS AND METHODS

### Drug

Cucurbitacin B was purchased from the National Institute for the Control of Pharmaceutical and Biological Products (Beijing, China), with a purity of 99.63%. SCH772984 was purchased from Ablome, USA. Compound C was purchased from Selleck Chemicals (Houston, TX).

### Cell culture

Human pancreatic cancer cell lines, ASPC-1, BxPC-3, CFPAC-1, HPAC and MiaPaCa-2, and the human normal pancreatic ductal epithelial cell line HPDE6-C7 were purchased from American Type Culture Collection (ATCC, Manassas, VA, USA) and cultured as previously described [43, 44]. Cell lines were tested for the presence of mycoplasma on a monthly basis using the PCR method described by Uphoff and Drexler [45]. Images are shown in Supplementary Figure 3.

### Cell viability assay

Effects of cucurbitacin B and SCH772984 alone or in combination on pancreatic cancer cell growth were measured using the MTT (3-[4,5-dimethylthiazol-2-yl]-2,5-diphenyltetrazolium-bromide, Sigma-Aldrich, St. Louis, MO, USA) reduction assay as previously described [46]. IC_50_ values were calculated as drug concentrations necessary to inhibit 50% of growth compared to untreated control cells. The extent and direction of CuB and SCH772984 cytotoxic interactions were determined by standard isobologram analyses and by evaluating combination index (CI) values, which were calculated using CompuSyn software (ComboSyn, Inc, Paramus, NJ, USA) where CI< 1, CI=1, and CI> 1 indicate synergistic, additive, and antagonistic effects, respectively, as previously described [46–48].

### Cell death and cell cycle progression

Cell death was determined by trypan blue exclusion. Cell cycle distribution was determined by PI staining followed by flow cytometry as previously described [48]. Dead cells were also recorded as PI^+^ events (Sub-G1 population).

### LDH release assay

Culture medium was collected and LDH activity was assessed using an LDH cytotoxicity assay kit (Nanjing Jiancheng Bioengineering Institute, China) according to the manufacturer’s protocol. LDH activity was quantified by measuring absorbance at 450 nm with a microplate reader (Biotek, USA). LDH release (%) = [OD_450_ (sample)-OD_450_ (low control)/OD_450_ (high control) -OD_450_ (low control)] × 100.

### Annexin V/PI staining

BxPC-3 and HPAC cells were treated with CuB and SCH772984 alone or in combination and subjected to flow cytometry analysis to determine drug-induced cell death using an annexin V-fluorescein isothiocyanate (FITC)/propidium iodide (PI) apoptosis kit (Beckman Coulter, Brea, CA, USA) and a FACS Calibur flow cytometer (Becton Dickinson, San Jose, CA, USA) as previously described [46, 49].

### Western blotting analysis

Western blotting was performed using polyvinylidene difluoride (PVDF) membranes (Thermo Fisher Inc., Rockford, IL, USA) and immunoblotted with anti-EGFR, -pEGFR (Y1173), -STAT3, -pSTAT3(Y705), -AKT (Abcam, Cambridge, MA, USA), -PARP, -ERK, -pERK (T202/204), -pS6 (S240/244), -CDK1, -pCDK1(Y15), -CyclinB1, -Bcl-2, -Bcl-xl, -Mcl-1, -Survivin, -Bim, -AMPKα and -pAMPKα (T172) (Cell Signaling Technology, Danvers, MA, USA), -pAKT(T308), -pAKT(S473) (Affinity Biologicals, USA), or β-actin antibodies (Sigma-Aldrich) as previously described [49]. Immunoreactive proteins were visualized using the Odyssey Infrared Imaging System (Li-Cor) as described by the manufacturer.

### shRNA knockdown of EGFR

pMD-VSV-G and delta 8.2 plasmids were gifts from Dr. Dong at Tulane University. Plasmid maps are shown in Supplementary Figure 2. EGFR and non-target control lentiviral vectors were purchased from Sigma-Aldrich. Transfection was performed using Lipofectamine 3000 reagent (Invitrogen, USA) according to the manufacturer’s instructions. Briefly, a lentivirus vector, pMD-VSV-G, and delta 8.2 were cotransfected into TLA-HEK293T cells, and the culture medium was harvested 72 h post-transfection. BxPC-3 and HPAC cells were transduced by adding virus supernatant and polybrene (Sigma-Aldrich) for 3 h. Cells were harvested 5 days after lentiviral infection and used for subsequent analysis.

### CRISPR knockdown of AMPKα

The lentiCRISPRv2 plasmid was derived from Feng Zhang (Addgene plasmid #52961 [50]). Guide RNAs (gRNAs) were designed using the CRISPR design tool (http://crispr.mit.edu). Non-target control (NTC) and AMPKα vectors were generated using Feng Zhang’s protocol, which is available on the Addgene website (www.addgene.org). Lentivirus production and transduction were performed as described, except that psPAX2 (from Didier Trono, Addgene plasmid #12260) was used instead of delta 8.2 [51]. The following gRNAs were used: AMPKα, 5′-AGTGCCATGCATATTCCCCC-3′, NTC, 5′-GCACTACCAGAGCTAACTCA-3′.

### Establishment of a mouse pancreatic cancer xenograft model

Female BALB/c nude mice (18–22 g) were purchased from Vital River Laboratories (Beijing, China). The animal study was conducted following internationally recognized guidelines and was approved by the Animal Research Committee of Norman Bethune College of Medicine, Jilin University. The HPAC xenograft model was generated as previously described [43]. When the xenografts reached a volume of 106.9 ± 13.4 mm^3^, mice were randomized into 4 groups (5 animals per group, with mean tumor volumes of 104.1 ± 7.7, 105.7 ± 7.3, 104.7 ± 8.3 and 115.2 ± 9.0 mm^3^ for the vehicle control, CuB, SCH772984, and combination group, respectively) and treated with (i) vehicle control, (ii) 0.5 mg/kg CuB three times per week by intraperitoneal injection, (iii) 25 mg/kg SCH772984 daily by intraperitoneal injection, or (iv) 0.5 mg/kg CuB three times a week by intraperitoneal injection and 25 mg/kg SCH772984 daily by intraperitoneal injection for 4 weeks. Tumor diameters were measured with a caliper daily. Mice were sacrificed after tumors in the control group reached 1000 mm^3^ by cervical vertebra dislocation. Tumor volume was calculated according to the following formula: m_1_^2^× m_2_ × 0.5236 (m_1_: short diameter; m_2_: long diameter). Tumor growth inhibition was calculated using the equation 100%×T/C, where C = final mean tumor volume – initial tumor volume for the control and T = final mean tumor volume – initial tumor volume for the treated groups.

### Hematoxylin and eosin, immunohistochemical and TUNEL staining

On day 28, mice were sacrificed and tumors from 5 mice in each treatment group were excised for hematoxylin and eosin (HE), proliferating cell nuclear antigen (PCNA) immunohistochemical and TUNEL staining. Immunohistochemical staining was analyzed using Motic Images Advanced 3.2 and expressed as the mean optical density (MOD). Five fields of vision without any overlap in each section were selected randomly and photographed at 200 magnification. Areas to measure were first detected using software, and integrated optical density of the target stain region was subsequently determined. The MOD from each slide reflected positive PCNA or TUNNEL expression. A higher MOD indicates increased positive expression.

### Statistical analysis

Data are expressed as the mean ± standard deviation of three experiments. Differences in the sample means between test groups and control groups were analyzed using the pair-wise two-sample *t*-test. Statistical analyses were performed with GraphPad Prism 5.0. *P* values less than 0.05 were considered significant and labeled as ^*^p < 0.05, ^**^p < 0.01, or ^***^p < 0.001.

## SUPPLEMENTARY MATERIALS FIGURES



## Abbreviations

PI: propidium iodide
H&E: hematoxylin and eosin
ip: intraperitoneal injection
T: mean treated tumor volume
C: control treated tumor volume
