# Supplementary material for: Cucurbitacin B and SCH772984 exhibit synergistic anti-pancreatic cancer activities by suppressing EGFR, PI3K/Akt/mTOR, STAT3 and ERK signaling
Source: Oncotarget. 2017 Oct 9;8(61):103167–81. doi: 10.18632/oncotarget.21704 (PMC5732720; doi:10.18632/oncotarget.21704)
Supplement: Supplementary file 1 [file oncotarget-08-103167-s001.pdf]

Cucurbitacin B and SCH772984 exhibit synergistic anti-pancreatic cancer activities by suppressing EGFR, PI3K/Akt/mTOR, STAT3 and ERK signaling

SUPPLEMENTARY MATERIALS

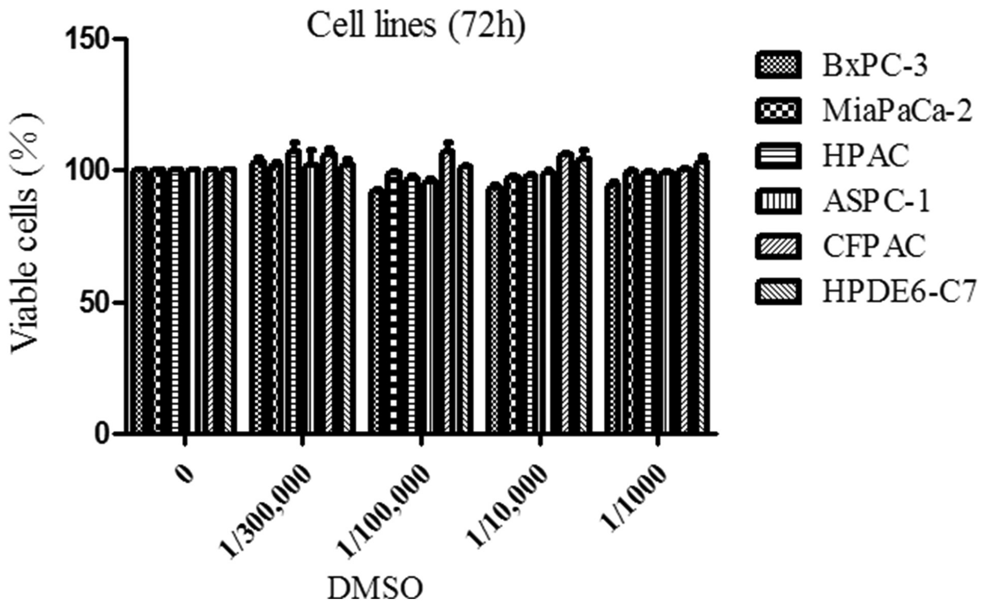

Supplementary Figure 1: Effect of DMSO on cell proliferation. BxPC-3, MiaPaCa-2, HPAC, ASPC-1, CFPAC and HPDE6-C7 cells were cultured with DMSO (ranging from 1/300,000 to 1/1000). Viable cells were determined with the MTT assay. Data are presented as the mean ± standard error from at least 3 independent experiments.

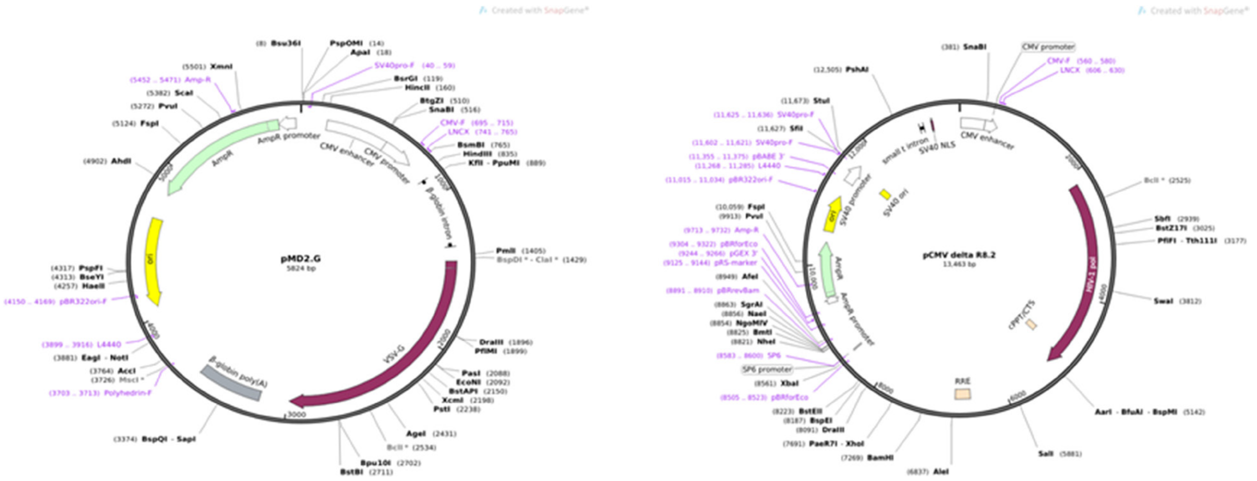

Supplementary Figure 2: Plasmid maps for pMD-VSV-G and delta 8.2. Plasmid maps for pMD-VSV-G and delta 8.2 are provided on the left and right, respectively.

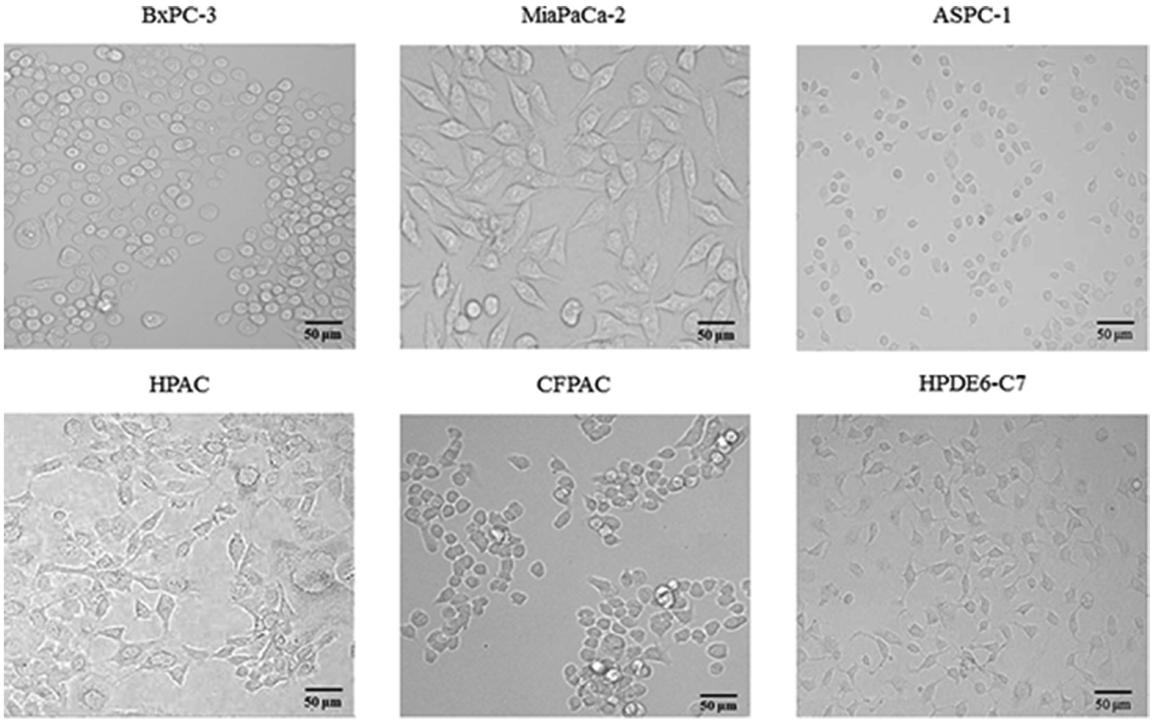

Supplementary Figure 3: Images of 6 cell lines. BxPC-3, MiaPaCa-2, HPAC, ASPC-1, CFPAC and HPDE6-C7 cells were viewed at ×200 magnification, scale bar = 50 µm.
